# Supplementary material for: Effects of High-Intensity Motor Learning and Dietary Supplementation on Motor Skill-Related Physical Fitness in Thin Ethiopian Children Aged 5 to 7 Years: An Exploratory Pilot Cluster-Randomized Trial
Source: Nutrients. 2024 Dec 25;17(1):30. doi: 10.3390/nu17010030 (PMC11723309; doi:10.3390/nu17010030)
Supplement: Supplementary file 1 [file nutrients-17-00030-s001.zip › nutrients-3360935-supplementary.pdf]

Annex tables

Program

During HiML training, intensity is determined by the amount of hours spent on the task activity, which is 5 times per week for 1 hour a day, over a total duration of 12 weeks and 60 hours of training.

Activity Categories, and Subactvity of HiML

A. Gross motor activity (subactivities are 1. Passing and controlling drills, 2. Shooting drills, 3. Passing, receiving & dribbling drills, 4. Dribbling, passing from right side, 5. Dribbling, passing from the left side, and receiving by use assistance, 6. Dribbling, 7. Dribbling between four cones, passing and receiving, 8. Dribbling, passing, and receiving 9. Dribbling, passing, receiving, and shooting, 10. Dribbling zigzag and Shooting, 11. Dribbling zigzag, passing to partner and Shooting, and 12. Dribbling between two cones, passing and receiving)

B. Playground activity (subactivities are: 1. Passing, receiving, and dribbling drills,2. Shooting to goal from triangle, 3.Shooting to the goal from the triangle through assistance, 4. Dribbling and turning over the cone, 5.Dribbling and turning over the player, 6.Dribbling and turning over the player and shooting, 7.Dribbling between four cones, passing and receiving, 8.Turning, dribbling between four cones, passing and receiving, and 9. Turning, dribbling between two cones, and shooting)

C.Sports ball (subactivities are: 1.1 vs 1 gameplay, 2.2 vs 2 gameplay, 3.3 vs 3 gameplay, 4.2 vs 4 gameplay, 5.3 vs 5 gameplay, 6.3 vs 3 children play ball, 7.4 vs 4 children play, and 8.4 vs 5 children play + Goalkeeper)

D. Cultural play (like hide and seek, hand dancing) (subactivities are :1. Rosa Rosina, 2. Shumbrarushe-ararushe, 3. Yes-ready, and 4. Oringo-papaya)

E.Cultural (singing, dancing) (Subactiviies are : 1.Pepsi , 2.Chipi-chipi papa , 3.Andi Lemendi , and 4.Dimbish le dimbish)

NB:

- Three activities are done every Monday, Tuesday, and Wednesday, except Thursday and Friday (four activities each).
- The total time spent on activities for each day is 1 hr (60 minutes).
- Recovery or rest between activity 5m; and Cooldown 5-10min
- The children rest for 2-3 minutes between each intensive activity.

Table S1. Description of the items of the PERFormance and FITness test battery (PERF-FIT)

**PERF-FIT**	
**Items**	**Description**
**Motor Skill Performance subscale**	
Bouncing and Catching (#)	Children bounce a tennis ball to the floor and catch it. This series involves five bouncing and catching items of increasing skill difficulty.
Throwing and Catching (#)	Children throw tennis ball in the air to at least eye level height and catch it. This series involves five throwing and catching items of increasing skill difficulty.
Jump (cm)	Children are asked to jump inside an agility ladder. This series involves four jumping items of increasing difficulty
Hop (#)	Children are asked to hop inside an agility ladder. This series involves four hopping items of increasing difficulty for each leg
Balance (s/#)	Children are asked to perform two static balance tasks for each leg and three dynamic balance tasks. Tasks involve knee hugging, grasping the foot and picking up cans from the floor
**Agility and Power Subscale**	
Running (s)	Children are asked to run (one foot per square) in 3.5m agility ladder and run around a bottle placed at a distance of 50cm and back.
Stepping (s)	Children step with two feet in each square of a 3.5m agility ladder and run around a bottle placed at a distance of 50cm and back
Side Jump (#)	Children are required to jump sideways in the agility ladder. The total number of correct landings in 15s is recorded.
Long Jump (cm)	Children are asked to jump forward as far as possible and land on their feet with a balanced landing.
Overhand Throw (cm)	Children kneel just behind a starting line and throw a sandbag (2kg) forward as far as possible.

Table S2. Protocol for the intervention

Intervention	Dose/day	Frequency	Duration	Systematic treatment	Compliance parameter	Considerations for Implementation	Responsible Person
RUSF	RUSF: 7 sachets/per child/week (1 per day). Follow national guidelinesfor SFP.<br>1 packet/day 100g/day<br><br>500 kcal<br>12.5 g Pro<br>31g Fat<br>42.7 g CHO*	Daily follow-up.	12 weeks	guidelines for OTP: if technical capacity and supplies (staff) are available / if health services are available.<br><br>Deworming<br>Amoxicillin<br>Vaccinations<br>Malaria treatment.	Absence of intrahousehold sharing of RUSF<br><br>Empty Sachet counting	It can be consumed directly from the package with no dilution, mixing, or<br><br>cooking necessary.	Research staff<br><br>School teachers<br><br>Health extension workers<br><br>Data collectors
HiML	5 times per week for 60 minutes	Daily	12 weeks		Number of HiML sessions attended by the child		Research staff<br><br>School teachers, parents<br><br>Health extension workers<br><br>Data collectors

Table S3: Differences in change between baseline and end-line measurements of muscular fitness and motor skills among 5-7 years of age MT children in Jimma Town, Southwest Ethiopia, 2023

Variables	Intervention type	N	Baseline	Endline	Difference	95%CI		P
			Mean ±SD	Mean ±SD	Mean ±SD	Lower Bound	Upper Bound	
Running total	No intervention	21	11.93±2.22	10.80±1.77	-1.05±1.60	-1.910	-0.203	0.235
	RUSF	16	11.44±3.92	10.96±2.30	-0.42±4.25	-2.986	2.153	
	RUSF+HiML	13	12.06±2.46	9.42± 1.38	-2.59±2.36	-4.417	-0.7799	
	Total	50	11.80±2.88	10.37±1.94	-1.20±2.96	-2.178	-0.226	
Stepping total	No intervention	21	21.76±4.08	21.58±5.77	0.19±3.28	-1.440	1.823	<0.001
	RUSF	18	24.37±4.81	19.05±3.28	-6.01±3.67	-8.134	-3.895	
	RUSF+ HiML	19	25.05±3.82	16.65±3.61	-8.20±3.47	-9.992	-6.417	
	Total	58	23.65±4.42	18.88±4.76	-4.49±5.03	-5.939	-3.050	
Side jump total	No intervention	21	14.90±3.08	15.62±2.69	0.71±2.00	-0.197	1.626	<0.001
	RUSF	21	14.09±3.34	16.68±3.24	2.66±2.31	1.615	3.717	
	RUSF+ HiML	25	15.20±2.48	19.76±3.45	4.56±3.44	3.139	5.980	
	Total	67	14.76±2.95	17.48±3.60	2.76±3.11	2.001	3.520	
Long jump total	No intervention	21	85.85±17.08	88.66±20.19	2.81±6.39	-0.102	5.721	<0.001
	RUSF	22	74.38±30.25	106.38±24.41	32.00±21.07	22.660	41.348	
	RUSF+ HiML	25	90.83±22.01	113.66±15.99	22.82±25.93	12.119	33.528	
	Total	68	83.97±24.42	103.58±22.59	19.61±23.13	14.014	25.212	
Overhand throw total	No intervention	21	126.55±28.70	135.02±33.05	8.47±11.94	3.0372	13.9056	0.050
	RUSF	22	116.56±50.84	147.45±37.89	22.12±30.14	8.3977	35.8404	
	RUSF+ HiML	25	131.47±24.79	157.51±22.04	26.04±26.95	14.9125	37.1675	
	Total	68	125.13±36.27	147.31±32.19	19.30±25.29	13.1345	25.4744	
Bounce and catch total	No intervention	21	24.85±12.24	22.38±13.54	-2.47±5.13	-4.813	-0.139	<0.001
	RUSF	22	23.91±15.41	34.50±11.46	9.09±7.58	5.643	12.546	
	RUSF+ HiML	25	21.96±14.05	42.00±4.85	20.04±11.40	15.33	24.747	
	Total	68	23.48±13.84	33.51±13.05	9.55±12.66	6.462	12.642	
Throw and catch total	No intervention	21	26.62±12.01	23.00±12.86	-3.62±5.75	-6.23	-1.002	<0.001
	RUSF	22	25.36±13.37	31.86±13.09	4.81±8.23	1.062	8.556	
	RUSF+ HiML	25	23.92±16.73	41.76±5.59	17.84±13.78	12.150	23.529	
	Total	68	25.22±14.17	32.76±13.22	7.03±13.47	3.743	10.316	

RUSF: Ready to Use Supplementary Food, HiML: High-Intensity Motor Learning, SD: standard deviation

References

1. Mehta NM, Corkins MR, Lyman B, Malone A, Goday PS, Carney L, Monczka JL, Plogsted SW, Schwenk WF, American Society for Parenteral and Enteral Nutrition (ASPEN) Board of Directors. Defining pediatric malnutrition: a paradigm shift toward etiology-related definitions. Journal of Parenteral and Enteral Nutrition. 2013 Jul;37(4):460-81.
2. UNICEF. Global Nutrition Report 2020. 2020. Available online: https://data.unicef.org/resources/global-nutrition-report-2020/.
3. World Health Organization. Fact sheets—malnutrition. World Health Organization [Internet]. 2024 March. Retrieved from: https://www.who.int/news-room/fact-sheets/detail/malnutrition
4. Saavedra JM, Prentice AM. Nutrition in school-age children: a rationale for revisiting priorities. Nutrition Reviews. 2023 Jul 1;81(7):823-43.

5. Hailegebriel T. Prevalence and determinants of stunting and thinness/wasting among schoolchildren of Ethiopia: a systematic review and meta-analysis. Food and Nutrition Bulletin. 2020 Dec;41(4):474-93.
6. Savarino G, Corsello A, Corsello G. Macronutrient balance and micronutrient amounts through growth and development. Italian journal of pediatrics. 2021 May 8;47(1):109.
7. Badaru UM, Umar AL, Abdullahi A, Usman JS, Ogwumike OO. Influence of malnutrition and body composition on the gross motor function of children with cerebral palsy in Kano, Nigeria: a cross-sectional study. Bulletin of Faculty of Physical Therapy. 2023 Jan 4;28(1):2.
8. Cichon B, Fabiansen C, Yaméogo CW, Rytter MJ, Ritz C, Briend A, Christensen VB, Michaelsen KF, Oummani R, Filteau S, Ashorn P. Children with moderate acute malnutrition have inflammation not explained by maternal reports of illness and clinical symptoms: a cross-sectional study in Burkina Faso. Bmc Nutrition. 2016 Dec; 2:1-0.
9. Saleem J, Zakar R, Bukhari GM, Fatima A, Fischer F. Developmental delay and its predictors among children under five years of age with uncomplicated severe acute malnutrition: a cross-sectional study in rural Pakistan. BMC Public Health. 2021 Dec; 21:1-0.
10. Isanaka S, Barnhart DA, McDonald CM, Ackatia-Armah RS, Kupka R, Doumbia S, Brown KH, Menzies NA. Cost-effectiveness of community-based screening and treatment of moderate acute malnutrition in Mali. BMJ global health. 2019 Apr 1;4(2): e001227.
11. Amegovu AK, Ogwok P, Ochola S, Yiga P, Musalima JH, Mutenyo E. Formulation of sorghum-peanut blend using linear programming for treatment of moderate acute malnutrition in Uganda. Journal of Food Chemistry and Nutrition. 2013 Oct 25;1(2):67-77.
12. Roche ML, Samson KL, Green TJ, Karakochuk CD, Martinez H. Perspective: Weekly iron and folic acid supplementation (WIFAS): A critical review and rationale for inclusion in the essential medicines list to accelerate anemia and neural tube defects reduction. Advances in Nutrition. 2021 Mar 1;12(2):334-42.
13. Lagrone L, Cole S, Schondelmeyer A, Maleta K, Manary MJ. Locally produced ready-to-use supplementary food is an effective treatment of moderate acute malnutrition in an operational setting. Annals of tropical pediatrics. 2010 Jun 1;30(2):103-8.
14. Training Module on the National Guidelines on the Management of Moderate Acute Malnutrition for Children under Five Years. Revised on 2020;(May). Available at: https://docs.wfp.org/api/documents/WFP-0000116054/download/.
15. Teshome MS, Lema TB, Abessa TG, et al. Current evidence on the effectiveness of Ready-to-Use Supplementary Foods in children with moderate acute malnutrition: a systematic review and meta-analysis. Journal of Nutritional Science. 2023;12: e130. doi:10.1017/jns.2023.114
16. Teshome MS, Verbecque E, Mingels S, Granitzer M, Abessa TG, Bruckers L, Belachew T, Rameckers E. Investigating the Effects of Dietary Supplementation and High-Intensity Motor Learning on Nutritional Status, Body Composition, and Muscle Strength in Children with Moderate Thinness in Southwest Ethiopia: A Cluster-Randomized Controlled Trial. Nutrients. 2024; 16(18):3118.
17. Teshome MS, Bekele T, Verbecque E, Mingels S, Granitzer M, Abessa TG, Lema TB, Rameckers E. Body composition and associated factors among 5–7-year-old children with

moderate acute malnutrition in Jimma town in southwest Ethiopia: A comparative cross-sectional study. Maternal & Child Nutrition. 2024 Jul;20(3):e13655.
18. Cruz AG, Suárez JF, Ciro JO, Chavarro NR, Villegas JG. Association between nutritional status and physical abilities in children aged 6–18 years in Medellin (Colombia). Anales de Pediatría (English Edition). 2014 Dec 1;81(6):343-51.
19. Oyhenart EE. Body composition in relation to nutritional status and socio-environmental conditions in schoolchildren living in the urban periphery of La Plata, Argentina. ALAN [online]. 2020, vol. 70, n. 2: 81-94https://doi.org/10.3390/nu16183118
20. Amit Bandyopadhyay, Sohini Basu. Motor Fitness in Children: A Brief Update. Res Inves Sports Med. 8(5), RISM.000699. 2022. DOI: 10.31031/RISM.2022.08.000699
21. Gao Z, Wen X, Fu Y, Lee JE, Zeng N. Motor skill competence matters in promoting physical activity and health. BioMed research international. 2021;2021. Available from: https://www.ncbi.nlm.nih.gov/pmc/articles/PMC8748756/
22. De P, Chattopadhyay N. Effects of malnutrition on child development: Evidence from a backward district of India. Clinical Epidemiology and Global Health. 2019 Sep 1;7(3):439-45.
23. Pienaar AE. Pienaar The association between under-nutrition, school performance and perceptual motor functioning in first-grade South African learners: The North-West Child Health Integrated with Learning and Development study. Health SA Gesondheid. 2019;24.
24. DiGirolamo AM, Ochaeta L, Flores RM. Early childhood nutrition and cognitive functioning in childhood and adolescence. Food and Nutrition Bulletin. 2020 Jun;41(1_suppl):S31-40.
25. Pranoto NW, Fauziah V, Muchlis AF, Komaini A, Rayendra R, Susanto N, Fitriady G, Setyawan H, Pavlovic R, Sibomana A, Ndayisenga J. Exploration of children'S motor skills with stunting vs. Non-stunting. Retos: nuevas tendencias en educación física, deporte y recreación. 2024(54):224-34.
26. National Research Council (US) and Institute of Medicine (US) Committee on Integrating the Science of Early Childhood Development. *From Neurons to Neighborhoods: The Science of Early Childhood Development*; Shonkoff, J.P., Phillips, D.A., Eds.; National Academies Press (US): Washington, DC, USA, 2000; ISBN 978-0-309-06988-5.
27. Bolger LE, Bolger LA, O'Neill C, Coughlan E, O'Brien W, Lacey S, Burns C, Bardid F. Global levels of fundamental motor skills in children: A systematic review. Journal of Sports Sciences. 2021 Apr 3;39(7):717-53.
28. Bornstein, M.H.; Britto, P.R.; Nonoyama-Tarumi, Y.; Ota, Y.; Petrovic, O.; Putnick, D.L. Child Development in Developing Countries: Introduction and Methods. *Child Dev.* 2012, *83*, 16–31.
29. Lubans, D.R.; Morgan, P.J.; Cliff, D.P.; Barnett, L.M.; Okely, A.D. Fundamental Movement Skills in Children and Adolescents: Review of Associated Health Benefits. *Sports Med.* **2010**, *40*, 1019–1035.
30. Subasinghe SM, Wijesinghe DG. The effect of nutritional status on cognitive and motor development of pre-school children.2006. Retrieved from: http://www.pgia.ac.lk/files/Annual_congress/journel/v18/8.pdf
31. Bull FC, Al-Ansari SS, Biddle S, Borodulin K, Buman MP, Cardon G, Carty C, Chaput JP, Chastin S, Chou R, Dempsey PC. World Health Organization 2020 guidelines on physical

activity and sedentary behaviour. British journal of sports medicine. 2020 Dec 1;54(24):1451-62.

32. Baran J, Weres A, Wyszyńska J, Pitucha G, Czenczek-Lewandowska E, Rusek W, Leszczak J, Mazur A. 60 minutes per day in moderate to vigorous physical activity as a natural health protector in young population. International Journal of Environmental Research and Public Health. 2020 Dec;17(23):8918.
33. Faigenbaum AD, Bruno LE. A fundamental approach for treating pediatric dynapenia in kids. ACSM's Health & Fitness Journal. 2017 Jul 1;21(4):18-24.
34. Moliner-Urdiales D, Ruiz JR, Ortega FB, Jiménez-Pavón D, Vicente-Rodriguez G, Rey-López JP, Martínez-Gómez D, Casajús JA, Mesana MI, Marcos A, Noriega-Borge MJ. Secular trends in health-related physical fitness in Spanish adolescents: the AVENA and HELENA studies. Journal of science and medicine in sport. 2010 Nov 1;13(6):584-8.
35. Myer G, Faigenbaum A, Ford K, Best T, Bergeron M, Hewett T. When to initiate integrative neuromuscular training to reduce sports-related injuries and enhance health in youth? Curr Sports Med Rep. 2011;10(3):155–66.
36. Robinson LE, Stodden DF, Barnett LM, Lopes VP, Logan SW, Rodrigues LP, D'Hondt E. Motor competence and its effect on positive developmental trajectories of health. Sports medicine. 2015 Sep; 45:1273-84.
37. Dipasquale V, Cucinotta U, Romano C. Acute malnutrition in children: pathophysiology, clinical effects and treatment. Nutrients. 2020 Aug 12;12(8):2413.
38. Hardy L, Reinten-Reynolds T, Espinel P, Zask A, Okely A. Prevalence and correlates of low fundamental movement skill competency in children. Pediatrics. 2012; 130(2):e390–8.
39. Lopes VP, Rodriques LP, Maia JA, Malina RM. Motor coordination as predictor of physical activity in childhood. Scand J Med Sci Sports. 2011;21(5):663–9.
40. Smith JJ, Eather N, Weaver RG, Riley N, Beets MW, Lubans DR. Behavioral correlates of muscular fitness in children and adolescents: a systematic review. Sports Medicine. 2019 Jun 1; 49:887-904.
41. Collins H, Booth J, Duncan A, Fawkner S. The effect of resistance training interventions on fundamentalmovement skills in youth: a meta-analysis. Sports Med. Open. 2019; 5:17.
42. De Meester A, Stodden D, Goodway J, True L, Brian A, Ferkel R, Haerens L. Identifying a motor proficiency barrier for meeting physical activity guidelines in children. Journal of science and medicine in sport. 2018 Jan 1;21(1):58-62.
43. Faigenbaum AD, MacDonald JP, Stracciolini A, Rebullido TR. Making a strong case for prioritizing muscular fitness in youth physical activity guidelines. Current Sports Medicine Reports. 2020 Dec 1;19(12):530-6.
44. Caspersen, C.J.; Powell, K.E.; Christenson, G.M. Physical Activity, Exercise, and Physical Fitness: Definitions and Distinctions for Health-Related Research. *Public Health Rep.* 1985, *100*, 126–131.
45. Goodway, J.D.; Ozmun, J.C.; Gallahue, D.L. *Understanding Motor Development: Infants, Children, Adolescents, Adults*; Jones & Bartlett Learning: Burlington, MA, USA, 2019; ISBN 978-1-284-17494-6.
46. Tortella P, Haga M, Loras H, Sigmundsson H, Fumagalli G. Motor skill development in Italian pre-school children induced by structured activities in a specific playground. PloS one. 2016 Jul 27;11(7):e0160244.

47. Jackman M, Lannin N, Galea C, Sakzewski L, Miller L, Novak I. What is the threshold dose of upper limb training for children with cerebral palsy to improve function? A systematic review. Austral Occup Ther J 2020; 67: 269–80.
48. Smits-Engelsman Bc, Blank R, Van Der Kaay Ac, Mosterd-Van Der Meijs Ri, Vlugt-Van Den Brand El, Polatajko HJ, Wilson PH. Efficacy of interventions to improve motor performance in children with developmental coordination disorder: a combined systematic review and meta-analysis. Developmental Medicine & Child Neurology. 2013 Mar;55(3):229-37.
49. Størvold GV, Jahnsen R. Intensive motor skills training program combining group and individual sessions for children with cerebral palsy. Pediatric Physical Therapy. 2010 Jul 1;22(2):150-9.
50. Zhang D, Soh KG, Chan YM, Bashir M, Xiao W. Effect of Functional Training on Fundamental Motor Skills Among Children: A Systematic Review.2023
51. Fu T, Zhang D, Wang W, Geng H, Lv Y, Shen R, Bu T. Functional Training Focused on Motor Development Enhances Gross Motor, Physical Fitness, and Sensory Integration in 5–6-Year-Old Healthy Chinese Children. Frontiers in Pediatrics. 2022 Jul 11; 10:936799.
52. Payne VG, Isaacs LD. *Human Motor Development: A Lifespan Approach*. New York, NY: Routledge. (2017). doi: 10.4324/9781315213040
53. Zheng Y, Ye W, Korivi M, Liu Y, Hong F. Gender differences in fundamental motor skills proficiency in children aged 3–6 years: A systematic review and meta-analysis. International Journal of Environmental Research and Public Health. 2022 Jul 7;19(14):8318.
54. Campbell MK, Piaggio G, Elbourne DR, Altman DG. Consort 2010 statement: extension to cluster randomized trials. Bmj. 2012 Sep 4;345.
55. EPHI I. Ethiopian Public Health Institute (EPHI)[Ethiopia] and ICF. Ethiopia Mini Demographic and Health Survey 2019: Key Indicators. 2019. Available from: https://dhsprogram.com/pubs/pdf/FR363/FR363.pdf
56. Gebreyesus SH, Lunde T, Mariam DH, Woldehanna T, Lindtjørn B. Is the adapted Household Food Insecurity Access Scale (HFIAS) developed internationally to measure food insecurity valid in urban and rural households of Ethiopia? BMC nutrition. 2015 Dec;1:1-0.
57. Coates J, Bilinsky P, Coates J. Household Food Insecurity Access Scale (HFIAS) for Measurement of Food Access : Indicator Guide version 3 Household Food Insecurity Access Scale (HFIAS) for Measurement of Food Access : Indicator Guide VERSION 3. 2007. Available from: https://www.fantaproject.org/sites/default/files/resources/HFIAS_ENG_v3_Aug07.pdf
58. NIHR, Biomedical Research Centre. procedure for Measuring the height of children over 2. 2017;(June):2–5. Available from: http://www.uhs.nhs.uk/Media/Southampton-Clinical-Research/Procedures/BRCProcedures/Procedure-for-height-of-children-over-2.pdf.
59. World Health Organization. WHO child growth standards: length/height-for-age, weight-for-age, weight-for-length, weight-for-height, and body mass index-for-age: methods and development. World Health Organization; 2006. Available from: http://www.who.int/childgrowth/standards/en/

60. Smits-Engelsman BC. PERFormance and FITness battery for children: PERF-FIT, manual. Cape Town. 2018.
61. Smits-Engelsman BC, Bonney E, Neto JL, Jelsma DL. Feasibility and content validity of the PERF-FIT test battery to assess movement skills, agility and power among children in low-resource settings. BMC Public Health. 2020 Dec; 20:1-1.
62. Smits-Engelsman B, Smit E, Doe-Asinyo RX, Lawerteh SE, Aertssen W, Ferguson G, Jelsma DL. Inter-rater reliability and test-retest reliability of the Performance and Fitness (PERF-FIT) test battery for children: a test for motor skill related fitness. BMC pediatrics. 2021 Dec;21(1):1-1.
63. Verbecque E, Coetzee D, Ferguson G, Smits-Engelsman B. High BMI and low muscular fitness predict low motor competence in school-aged children living in low-resourced areas. International journal of environmental research and public health. 2021 Jul 25;18(15):7878.
64. Deutz NE, Ashurst I, Ballesteros MD, Bear DE, Cruz-Jentoft AJ, Genton L, Landi F, Laviano A, Norman K, Prado CM. The underappreciated role of low muscle mass in the management of malnutrition. Journal of the American Medical Directors Association. 2019 Jan 1;20(1):22-7.
65. Azimi F, Esmaillzadeh A, Alipoor E, Moslemi M, Yaseri M, Hosseinzadeh-Attar MJ. Effect of a newly developed ready-to-use supplementary food on growth indicators in children with mild to moderate malnutrition. Public Health. 2020 Aug 1; 185:290-7.
66. Steenkamp L, Lategan R, Raubenheimer J. The impact of Ready-to-Use Supplementary Food (RUSF) in targeted supplementation of children with moderate acute malnutrition (MAM) in South Africa. South African Family Practice. 2015 Sep 3;57(5):322-5.
67. McDonough DJ, Liu W, Gao Z. Effects of physical activity on children's motor skill development: a systematic review of randomized controlled trials. BioMed research international. 2020;2020(1):8160756.
68. Sutapa P, Pratama KW, Rosly MM, Ali SK, Karakauki M. Improving motor skills in early childhood through goal-oriented play activity. Children. 2021 Nov 2;8(11):994.
69. Han A, Fu A, Cobley S, Sanders RH. Effectiveness of exercise intervention on improving fundamental movement skills and motor coordination in overweight/obese children and adolescents: A systematic review. Journal of science and medicine in sport. 2018 Jan 1;21(1):89-102
70. Wang X, Zhou B. Motor development-focused exercise training enhances gross motor skills more effectively than ordinary physical activity in healthy preschool children: an updated meta-analysis. Frontiers in Public Health. 2024 May 21; 12:1414152.
71. Tinderholt Myrhaug H, Østensjø S, Larun L, Odgaard-Jensen J, Jahnsen R. Intensive training of motor function and functional skills among young children with cerebral palsy: a systematic review and meta-analysis. BMC pediatrics. 2014 Dec; 14:1-9.
72. Draper CE, Achmat M, Forbes J, Lambert EV. Impact of a community-based programme for motor development on gross motor skills and cognitive function in preschool children from disadvantaged settings. Early child development and care. 2012 Jan 1;182(1):137-52.
73. Moghaddaszadeh A, Belcastro AN. Guided active play promotes physical activity and improves fundamental motor skills for school-aged children. Journal of sports science & medicine. 2021 Mar;20(1):86.

74. Fjørtoft I. Motor fitness in pre-primary school children: the EUROFIT motor fitness test explored on 5–7-year-old children. Pediatric exercise science. 2000 Nov 1;12(4):424-36.
75. Ning K, Shen X, Shao X. Research on the age and gender characteristics of the development of gross motor in preschool children. Chinese Journal of Child Health Care. 2016;24(12):1322-5.
76. Sheikh M, Safania AM, Afshari J. Effect of selected motor skills on motor development of both genders aged 5 and 6 years old. Procedia-Social and Behavioral Sciences. 2011 Jan 1; 15:1723-5.
77. Yang SC, Lin SJ, Tsai CY. Effect of sex, age, and BMI on the development of locomotor skills and object control skills among preschool children. Perceptual and motor skills. 2015 Dec;121(3):873-88.
78. Lee J, Zhang T, Chu TL, Gu X. Effects of a need-supportive motor skill intervention on children's motor skill competence and physical activity. Children. 2020 Mar 17;7(3):21.
79. Hurtado-Almonacid J, Reyes-Amigo T, Yáñez-Sepúlveda R, Cortés-Roco G, Oñate-Navarrete C, Olivares-Arancibia J, Páez-Herrera J. Development of Basic Motor Skills from 3 to 10 Years of Age: Comparison by Sex and Age Range in Chilean Children. Children. 2024 Jun 11;11(6):715.
80. Hemming K, Taljaard M, Gkini E, Bishop J. Sample size determination for external pilot cluster randomised trials with binary feasibility outcomes: a tutorial. Pilot and Feasibility Studies. 2023 Sep 19;9(1):163.
81. Abessa TG, Worku BN, Wondafrash M, Girma T, Valy J, Lemmens J, Bruckers L, Kolsteren P, Granitzer M. Effect of play-based family-centered psychomotor/psychosocial stimulation on the development of severely acutely malnourished children under six in a low-income setting: a randomized controlled trial. BMC pediatrics. 2019 Dec;19:1-20.
82. Gericke C, Pienaar AE, Gerber B, Monyeki MA. Relationships between moderate vigorous physical activity, motor-and health-related fitness and motor skills in children. African Journal of Primary Health Care & Family Medicine. 2024 May 20;16(1):4258.
